# Cultivating Physical and Mental Wellness: The Impact of Storytelling and Puzzle Sessions on Generational Change

**DOI:** 10.7759/cureus.67598

**Published:** 2024-08-23

**Authors:** Rajeswari Kathiah

**Affiliations:** 1 Pathology, All India Institute of Medical Sciences (AIIMS), Madurai, IND

**Keywords:** problem-solving skills, youth engagement, community initiative, generational change, mental wellness, physical wellness, puzzle sessions, storytelling

## Abstract

In the urban landscape of Chennai, a pathologist, deeply concerned about the future of the younger generation, initiated a community-driven initiative aimed at fostering physical and mental wellness among children. Through a blend of captivating storytelling and stimulating puzzle sessions, the initiative sought to combat trends of laziness, gadget dependency, and poor dietary habits prevalent among youth. The sessions, designed to engage young minds while imparting valuable life lessons and problem-solving skills, garnered remarkable responses from participants and parents alike. Children previously disengaged or branded as slow learners or attention deficit by their peers and parents also displayed increased curiosity and attentiveness, underscoring the efficacy of interactive learning experiences. Lessons gleaned from the initiative highlighted the innate love for stories, the joy of puzzles, the impact of shared experiences, parental influence, the importance of healthy habits, moral education, and the listening skills of children. This conclusion was derived based on the consistent observations made during the sessions, where children's increased attentiveness and participation, particularly those with attention deficit, were noted through qualitative assessments and feedback from parents, alongside informal behavioral evaluations conducted throughout the initiative. The journey not only transformed the initiator into a nurturing storyteller but also influenced her role as a mother, emphasizing genuine interaction and empathetic communication. The call to action emphasizes proactive measures to instill positive habits and values in children, prioritizing holistic development over traditional measures of success. By investing in storytelling, puzzle-solving, and empathetic mentorship, stakeholders can empower future generations to navigate life's challenges with resilience and compassion. Ultimately, the power of storytelling lies in its ability to captivate, inspire, and shape the hearts and minds of individuals across generations, paving the way for a brighter tomorrow.

## Editorial

Introduction

In the bustling city of Chennai, amidst the daily hustle and bustle, I, a pathologist by profession, found myself deeply concerned about the future of the younger generation. Living within a close-knit gated community for the past decade, I couldn't help but notice the pervasive trends of laziness, irresponsibility, and a concerning dependency on gadgets and junk food among children. My professional life, characterized by daily encounters with the stark realities of disease pathology, further fueled my determination to contribute positively to society. Thus, armed with a passion for storytelling and puzzle creation, I embarked on a journey to inspire and nurture the children within my community [1].

Engagement initiatives

With a firm belief in the transformative power of storytelling, I initiated weekend sessions within our community premises. The age of the children was from seven to 14 years and consisted of both boys and girls. The number of children per session varied between 20 and 25 based on their availability. No child missed more than one consecutive session. Sessions were attended only by the children without being accompanied by their parents/guardians/caretakers. These sessions that lasted for one hour were meticulously crafted to engage young minds through a blend of captivating narratives and stimulating puzzles.

Each session commenced with an enchanting tale carefully selected to impart valuable life lessons. The majority of the stories were adapted from online sources and tailored by the author to align with the children's interests. As the children eagerly listened, their imaginations soared, transporting them to worlds unknown. Following the stories, they delved into many puzzles, each designed to challenge their intellect and foster problem-solving skills. Puzzles were both word- and picture-based.

Moreover, interspersed throughout the sessions were real-life anecdotes, inspired by my experiences in pathology as I learn from every patient who attends our hematology, cytology, and histopathology laboratories. The life lessons imparted during the sessions were carefully woven into the stories, which were both personal and adapted from various moral stories found on the internet. For instance, I often drew from my own experiences as a pathologist, sharing real-life anecdotes about the importance of health, empathy, and resilience that I had encountered through my patients. One memorable story involved a patient who overcame severe illness through perseverance and a positive mindset, which resonated deeply with the children and emphasized the power of mental strength in difficult times.

Additionally, unique moral stories sourced online were carefully chosen to suit the children's age group and were shared with deep emotion, making them relatable and impactful. Stories like "The Boy Who Cried Wolf" were told not just for their moral value but with added layers of empathy, highlighting the consequences of dishonesty in a compassionate light. Another story involved a king who learned the importance of humility and kindness from a poor villager, demonstrating to the children that true strength lies in character, not in material wealth. The combination of real-life experiences and these carefully selected moral stories helped foster emotional connections and instilled essential life values in the children. These sessions continued without interruption for a period of three years, occurring monthly.

Remarkable Responses

The response from the children exceeded all expectations. What began as a modest endeavor quickly blossomed into a vibrant community initiative, with children eagerly anticipating each session. Attendance became contingent upon a week-long commitment to abstain from gadgets and junk food, a requirement enthusiastically embraced by both children and parents alike. Even children previously labeled as "slow learners" or branded as attention deficit also exhibited remarkable curiosity and attentiveness during the sessions. It was a testament to the power of engaging storytelling and interactive learning experiences.

For example, few children, who were previously labeled as slow learners, initially struggled to engage with the stories and puzzles. However, after a few sessions, I noticed a marked change in their behavior. They began asking thoughtful questions about the characters and plots, showing a genuine curiosity to understand more. Additionally, during the puzzle activities, the children displayed increased focus, managing to complete more complex puzzles with minimal guidance, something they were unable to do when they first joined the sessions. These examples highlight the positive impact that storytelling and puzzles had on fostering attentiveness and curiosity in children facing attention deficits.

The assessment of the initiative's impact was not conducted through formal methods but rather through careful observation and ongoing feedback from the parents. I closely monitored the children's behavior and engagement during the sessions, noting significant improvements in attentiveness, problem-solving abilities, and social interactions, particularly in children previously diagnosed with ADHD. In addition to my observations, parents frequently provided informal feedback, reporting positive changes in their children's habits at home, including reduced gadget dependency, better focus, and increased enthusiasm for learning. This combination of careful observation and parental feedback provided valuable insights into the success of the storytelling and puzzle sessions, without the need for formal assessments.

Lessons Learned from Children

Through this enriching journey, the children imparted invaluable lessons of their own:

Love for stories: Children possess an innate love for stories, provided they are delivered with care, patience, and skill.

Joy of puzzles: The joy of puzzles lies not only in their solution but also in the process of discovery. Offering a diverse array of puzzles ensures sustained interest and cognitive growth.

Impact of shared experiences: Emotional sharing of experiences fosters deep connections and immersive learning, leading to lasting behavioral changes.

Parental influence: The dissatisfaction often observed in children stems from parental neglect, emphasizing the importance of quality time and genuine engagement over material possessions.

Healthy habits: Children mirror the behaviors modeled by their parents, underscoring the need for positive role modeling in dietary habits and lifestyle choices.

Moral education: Moral values are best internalized through the art of storytelling, which resonates deeply with children and leaves a lasting impression.

Listening skills: Contrary to popular belief, children possess remarkable listening skills when engaged effectively and provided with a nurturing environment.

This conclusion was derived based on the consistent observations made during the sessions, where children's increased attentiveness and participation, particularly those with attention deficit, were noted through informal assessments and feedback from parents, alongside informal behavioral evaluations conducted throughout the initiative.

Discussion

The findings from this initiative highlight the significant impact of storytelling and puzzle-based learning on children's cognitive and emotional development. Through careful observation and parental feedback, it became evident that these methods not only fostered curiosity and attentiveness but also enhanced social-emotional skills, problem-solving abilities, and behavioral improvements. These results align with previous studies that emphasize the role of interactive and narrative-based learning in promoting intellectual engagement, emotional regulation, and the development of life skills. The informal yet consistent improvements observed in attention-deficit children further underscore the value of these approaches in diverse educational settings.

Previous studies have demonstrated numerous benefits of both storytelling and puzzle-based learning in the cognitive and emotional development of children. Storytelling has been shown to enhance children’s social-emotional skills by fostering empathy, improving emotional regulation, and enhancing their ability to connect with others. Similarly, puzzle-based learning has been found to improve cognitive functions such as problem-solving, logical thinking, and spatial reasoning. According to Landrum et al., "puzzle-based learning helps enhance cognitive engagement by encouraging children to tackle challenges systematically [2]. It promotes perseverance and patience, as children learn to navigate through puzzles using strategic thinking and trial-and-error approaches. These activities also help build executive functions like working memory and attention, which are crucial for both academic performance and everyday tasks."

In comparison to similar studies that have formally employed psychometric tests to assess cognitive and emotional development in children, this initiative shared similar outcomes in enhancing problem-solving skills and emotional engagement, despite not utilizing standardized tests. However, unlike some studies that have measured cognitive development and found similar improvements in children's intellectual engagement, this initiative relied on organic observation and feedback rather than standardized testing, which may present limitations in quantifiable data but also allowed for more personalized insights into each child’s behavioral changes. The dissimilarity lies in the absence of formal testing here, yet the alignment in observed behavioral outcomes suggests that the initiative had a significant positive impact on the children’s mental wellness and cognitive development.

Personal Reflections

Engaging in these storytelling sessions not only influenced the children I worked with but also made me a better mother for my own children. I became more patient, learning to give them the space to express themselves and process their emotions without rushing them. For example, when my child would struggle with a task or feel frustrated, I applied the patience I had developed during the sessions, allowing them time to work through their feelings. Additionally, I became a better listener. Just as I had to listen carefully to the children's feedback during the sessions, I found myself listening more attentively to my own children’s stories and concerns, fostering a deeper connection with them. Rather than assigning blame, it underscored the need for collective action and investment in the holistic development of our children.

Call to Action

Consider the ramifications of unhealthy lifestyle choices and inadequate emotional management in the younger generation. If left unchecked, these behaviors can lead to detrimental genetic mutations, setting a troubling precedent for future generations. No amount of financial prosperity or healthcare policies can fully reverse the damage caused by these genetic alterations. What's more concerning is that these negative genetic changes have the potential to be passed down to successive generations, perpetuating a cycle of health issues [3].

As stewards of the future, we must recognize the profound impact of our actions on the genetic predispositions of generations to come. By embracing the roles of storytellers, puzzle masters, and empathetic mentors, we can empower our children to become resilient, compassionate, and intellectually curious individuals. Let us seize this opportunity to transform complaint boxes into catalysts for positive change, one story, one puzzle, and one heartfelt conversation at a time.

Therefore, we must take proactive measures to instill positive habits and values in our children from an early age. Instead of solely focusing on traditional measures of success, such as becoming doctors, scientists, or engineers, we must prioritize the development of essential life skills, such as effective communication and active listening. By fostering a culture of empathy, understanding, and emotional intelligence, we can empower our children to navigate life's challenges with resilience and grace [4].

Let us not underestimate the significance of our actions in shaping the genetic legacy of future generations. By investing in the holistic well-being of our children today, we can pave the way for a healthier, happier tomorrow. It is time to shift our focus from mere academic achievements to cultivating compassionate, empathetic individuals who are capable of making positive contributions to society [5].

Conclusion

In a world fraught with challenges, the power to effect meaningful change lies within our grasp. Through the simple yet profound acts of storytelling, puzzle-solving, and empathetic listening, we can cultivate a future generation equipped with the resilience and empathy needed to navigate life's complexities. Let us embark on this journey of transformation, guided by the wisdom and lessons imparted by our children, and together, build a brighter tomorrow for generations to come.

The power of storytelling lies in its ability to captivate and inspire. When stories are crafted with care and imbued with meaningful morals, they naturally resonate with listeners, regardless of age. By weaving engaging narratives that convey important life lessons, we not only entertain but also educate and instill values in our audience. Through this age-old tradition of storytelling, we have the opportunity to shape the hearts and minds of future generations, fostering empathy, resilience, and a deep appreciation for the richness of human experience.
